# Dynamic Stabilisation in the Treatment of Degenerative Disc Disease with Modic Changes

**DOI:** 10.1155/2013/806267

**Published:** 2013-05-20

**Authors:** Olcay Eser, Cengiz Gomleksiz, Mehdi Sasani, Tunc Oktenoglu, Ahmet Levent Aydin, Yaprak Ataker, Tuncer Suzer, Ali Fahir Ozer

**Affiliations:** ^1^Department of Neurosurgery, School of Medicine, Afyon Kocatepe University, Afyonkarahisar, Turkey; ^2^Department of Neurosurgery, Ordu Medical Park Hospital, Ordu, Turkey; ^3^Department of Neurosurgery, American Hospital, Istanbul, Turkey; ^4^Neurosurgery Department, Istanbul Physical Therapy and Rehabilitation Hospital, Istanbul, Turkey; ^5^Physical Therapy and Rehabilitation Department, American Hospital, Istanbul, Turkey; ^6^Department of Neurosurgery, School of Medicine, Koc University, Rumelifeneri Yolu Sarıyer, Istanbul 34450, Turkey

## Abstract

*Objective*. Posterior dynamic stabilization is an effective alternative to fusion in the treatment of chronic instability and degenerative disc disease (DDD) of the lumbar spine. This study was undertaken to investigate the efficacy of dynamic stabilization in chronic degenerative disc disease with Modic types 1 and 2. Modic types 1 and 2 degeneration can be painful. Classic approach in such cases is spine fusion. We operated 88 DDD patients with Modic types 1 and 2 via posterior dynamic stabilization. Good results were obtained after 2 years of followup. *Methods*. A total of 88 DDD patients with Modic types 1 and 2 were selected for this study. The patients were included in the study between 2004 and 2010. All of them were examined with lumbar anteroposterior (AP) and lateral X-rays. Lordosis of the lumbar spine, segmental lordosis, and ratio of the height of the intervertebral disc spaces (IVSs) were measured preoperatively and at 3, 12, and 24 months after surgery. Magnetic resonance imaging (MRI) analysis was carried out, and according to the data obtained, the grade of disc degeneration was classified. The quality of life and pain scores were evaluated by visual analog scale (VAS) score and Oswestry Disability Index (ODI) preoperatively and at 3, 12, and 24 months after surgery. Appropriate statistical method was chosen. *Results*. The mean 3- and 12-month postoperative IVS ratio was significantly greater than that of the preoperative group (*P* < 0.001). However, the mean 1 and 2 postoperative IVS ratio was not significantly different (*P* > 0.05). Furthermore, the mean preoperative and 1 and 2 postoperative angles of lumbar lordosis and segmental lordosis were not significantly different (*P* > 0.05). The mean VAS score and ODI, 3, 12, and 24 months after surgery, decreased significantly, when compared with the preoperative scores in the groups (*P* = 0.000). *Conclusion*. Dynamic stabilization in chronic degenerative disc disease with Modic types 1 and 2 was effective.

## 1. Introduction

Chronic low back pain (LBP) has been one of the most common causes of disability in adults and is a very important disease for early retirement in industrialized societies. Degenerative disc disease (DDD) is the most frequent problem in patients with LBP. The prevalence of Modic changes among patients with DDD of the lumbar spine varies between 19% and 59%. Type 1 and 2 Modic changes are more common than type 3 and mixed changes [1–13].

Degenerative vertebral endplate and subchondral bone marrow changes were first noted on magnetic resonance imaging (MRI) by Roos et al. in 1987 [1]. A formal classification was subsequently provided by Modic et al. in 1988, based on a study of 474 patients, most of whom had chronic LBP [2]. They were found to be associated with DD [1–3]. Three different types have been described [2, 3]. Type I lesions (low T1 and high T2 signals) are assumed to indicate an ongoing active degenerative process. Type II lesions (high T1 and T2 signals) are thought to manifest a more stable and chronic degeneration. Type III lesions (low T1 and T2 signals) are associated with subchondral bone sclerosis. Modic changes are interesting because an association between Modic changes and LBP symptoms has been shown recently in population-based cohorts [10, 12, 14].

Kjaer et al. suggested that Modic changes constitute the crucial element in the degenerative process around the disk in relation to LBP and clinical findings [14]. They demonstrated that DDD on its own was a fairly quiet disorder, whereas DDD with Modic changes was much more frequently associated with clinical symptoms. Most authors agree that among Modic changes, type 1 changes are those that are most strongly associated with symptomatic LBP [5, 7, 12, 13]. Braithwaite et al. suggested that vertebral endplate could be a possible source of discogenic LBP [4]. Therefore, Modic changes appear to be a relatively specific but insensitive sign of a painful lumbar disc in patients with discogenic LBP.

Buttermann et al. suggested that abnormal endplates associated with inflammation are a source of pain, and treating endplates directly with anterior fusion may be a preferred treatment for this subset of degenerative patients [15]. Chataigner et al. suggested that anterior fusion is effective for the treatment of LBP due to DDD when associated with vertebral plate changes [16]. Fritzell et al. reported that posterior lumbar fusion in patients with severe chronic LBP can diminish pain and decrease disability more efficiently than commonly used nonsurgical treatment, through a prospective multicenter randomized controlled trial from the Swedish Lumbar Spine Study Group [17]. Kwon et al. suggested that PLIF procedures in which TFC is used in patients with Modic types 1 and 2 showed an acceptably high success and fusion rate [18].

Segmental fusion operations are performed frequently as treatment for DDD with Modic types 1 and 2. Nevertheless, fusion also carries various risks such as adjacent segment degeneration, bone graft donor place pain, and pseudoarthrosis [19–22]. Dynamic stabilization controls abnormal movements in an unstable, painful segment and facilitates healthy load transfer, preventing degeneration of the adjacent segment [23]. Recently, several clinical studies reported that dynamic stabilization yielded good clinical results and represented a safe and effective alternative technique to spine arthrodesis in selected cases of degenerative lumbar spine instability [24–26].

The purpose of the current study was to assess the efficacy of dynamic stabilization in DDD with Modic types 1 and 2.

## 2. Materials and Methods

A total of 88 DDD patients with Modic types 1 and 2 were selected for this study. The patients were included in the study between 2004 and 2010. Among them, 70 patients showed Modic type 1 (80%) and 18 patients exhibited Modic type 2 (20%). The study patients consisted of 30 males and 58 females, with a mean age of 45 years (range: 25–65 years). All the patients received surgery, with 59 patients at L4-5 level (67%), 22 patients at L5-S1 level (25%), and 7 patients at L3-4 level (8%). Furthermore, 23 patients had (26 %) grade 3 and 65 patients had (74%) grade 4 disc degeneration.

Patients were informed about the operation. All the patients completed the consent forms. The patients had leg and/or chronic LBP, and those who had previously undergone spinal surgery were excluded. We also excluded patients with spinal tumor, infection, spondylolisthesis, traumatic vertebral fracture, scoliosis, and serious systemic disease. Patients were diagnosed to have DDD with Modic changes on MRI. All patients were examined with lumbar anteroposterior (A-P) and lateral X-rays. *Cosmic* (Ulrich GmbH & Co. KG, Ulm, Germany) and *Safinaz* (Medikon* AS*, Turkey) dynamic pedicle screws and rigid rod system were used together with the microdiscectomy procedure in all patients.

### 2.1. Evaluation of Quality and Pain Scores

The quality of life and pain scores were evaluated using visual analog scale (VAS) score (0, no pain; 10, worst pain) and Oswestry Disability Index (ODI) both preoperatively and at 3, 12, and 24 months after surgery (Table 2).

### 2.2. Radiological Analysis

The patients underwent preoperative MRI and/or computed tomography (CT). Furthermore, all patients had AP and lateral standing X-rays of the lumbar spine preoperatively and at 3 (1 postoperative), 12 (2 postoperative), and 24 months (3 postoperative) after surgery. Lordosis of the lumbar spine (L1-S1) was measured as the angle between the lines drawn on lateral standing X-rays from the lower endplate of L1 and upper endplate of S1. Segmental lordosis of the operative level (or levels) was measured as the angle between lines drawn from the upper and lower endplates of the vertebrae across which instrumentation spanned preoperatively as well as 3, 12, and 24 months after surgery. The ratio of the height of the intervertebral disc spaces (IVSs) to the vertebral body height was measured and compared preoperatively and postoperatively. The IVS ratio was calculated as the mean anterior and posterior intervertebral disc height divided by the vertebral height of the rostral vertebra of the motion segment.

### 2.3. MRI Evaluation

Lumber sagittal MRI was performed with a slice of 5 mm thickness. A T2-weighted image with a repetition of 2500 msec and an echo time of 90 msec of the lumbar spine was taken for all the participants. The signal intensity of nucleus pulposus of the discs L2-L3, L3-L4, L4-L5, and L5-S1 was evaluated independently by three radiologists. The grade of disc degeneration was determined according to Schneiderman's classification: Grade 1, normal signal intensity; Grade 2, heterogeneous decreased signal intensity; Grade 3, diffuse loss of signal; Grade 4, signal void. MRI analysis was carried out, and according to the data obtained, the grade of disc degeneration was classified as mild (Grades 1-2), and severe (Grades 3-4).

In this study, before surgery, endplate abnormalities were divided into Modic type 1 signals (low intensity on T1-weighted spin-echo images and high intensity on T2-weighted spin-echo images) and Modic type 2 signals (high intensity on both T1- and T2-weighted spin-echo images).

### 2.4. Operative Technique

**Figure 1:**
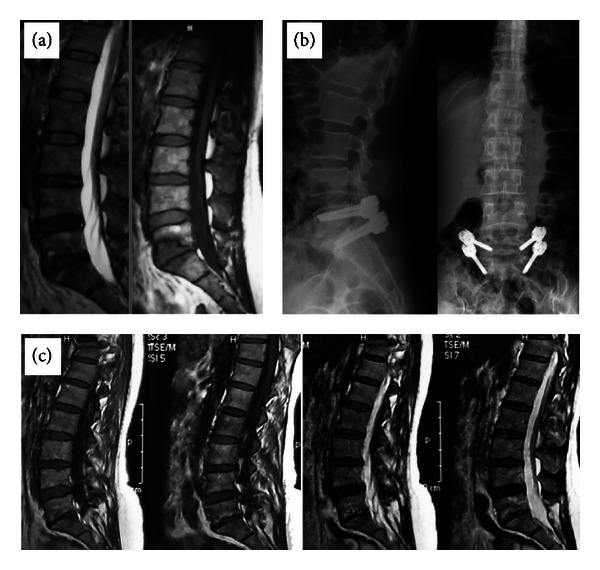
A 43-year-old female patient complained of severe back pain, particularly when standing or walking. (a) T1- and T2-weighted images showing hypointense corpus changes in upper and lower endplates. (b) Dynamic stabilization carried out with Safinaz screws. (c) T1- and T2-weighted MR images showing degenerative changes that shifted to Modic type 3, 2 years later.

All patients were taken into the operating room under general anesthesia in the prone position. Prophylactic antibiotics were given to all of them before the operation. All operations were performed using operational microscopy and standard surgical technique. The level of operation was determined via intraoperative fluoroscopy. When the interlaminar level with disc herniation was approached from the medial aspect, laminotomy was widened with the help of a high-speed drill. After identifying the correct nerve root, free disc fragments under the nerve root and passageway were removed. Decompression was completed by performing the required laminotomy. After carrying out the microdecompression procedure, we also executed posterior dynamic transpedicular stabilization from the same incision with the help of lateral intraoperative fluoroscopy using Wiltse approach via inside lateral paravertebral muscle. The dynamic pedicle hinged screws used in our cases were Cosmic (Ulrich Gmbh & Co. KG, Ulm, Germany) and Safinaz (Medikon,Turkey), in combination with rigid rods (Figure 1).

### 2.5. Statistical Methods

Kolmogorov-Smirnov test was used for homogeneity of the groups to comply with the normal distribution test. Friedman and Wilcoxon test was used for statistical analysis.

## 3. Results

In Table 1, the median, minimum and maximum range, Lumbar lordosis, *α* angle, and IVS value are given. The mean 1, 2, and 3 postoperative IVS ratio was significantly greater than that of the preoperative group (*P* < 0.001, Table 1). However, the mean 1 and 2 postoperative IVS ratio was not significantly different (*P* > 0.05). The mean preoperative and 1, 2, and 3 postoperative angles of lumbar lordosis and segmental lordosis were not significantly different (*P* > 0.05). Furthermore, the mean lumbar lordosis preoperative and 1, 2, and 3 postoperative values were not significantly different (*P* > 0.05).

All cases of Modic type 1 degeneration upgraded to type 2 or 3 degeneration after 24 months without pain.

From Table 2, it can be noted that the mean VAS pain score and ODI score 3, 12, and 24 months after surgery decreased significantly, when compared with the preoperative scores in the groups (*P* = 0.000). Furthermore, 24 months after surgery, the mean VAS score and ODI score decreased significantly, when compared with preoperative scores and postoperative 3- and 12-month scores in the groups (*P* = 0.000).

## 4. Discussion

Abnormalities of the vertebral endplate and vertebral bone marrow were described by Modic et al. [2]. Abnormalities associated with decreased signal intensity on T1-weighted spin-echo images (Modic type 1) correlated with segmental hypermobility and LBP [3]. Fayad et al. found that patients with chronic LBP and predominantly type 1 inflammatory Modic changes had better short-term relief of symptoms following intradiscal steroid injection than those with predominantly type 2 changes, which further supports the inflammatory nature of Modic type 1 changes and the role of inflammation in the generation of LBP [27]. Two recent publications suggest a possible relationship between bone marrow abnormalities revealed by MRI and discogenic pain [4, 28]. In these studies, moderate and severe types 1 and 2 endplate abnormalities were considered abnormal, and all the tested discs caused concordant pain on provocation [6]. Ohtori et al. reported that endplate abnormalities in patients with discogenic pain are related to inflammation and axonal growth into the abnormal bone marrow induced by cytokines, such as tumor necrosis factor-*α* [29]. Thus, tumor necrosis factor-*α* expression and sensory nerve in-growth in abnormal endplates may be a cause of LBP [29].

It has been reported that Modic type 1 change is associated with pathology, including disruption and fissuring of the endplate with regions of degeneration and regeneration and vascular granulation tissue [2, 5]. In addition, an increased amount of reactive woven bone as well as prominent osteoclasts and osteoblasts has been observed [2]. It has been reported that there were increases in the amount of cytokines and the density of sensory nerve fibers in the endplate and bone marrow in Modic type 1 change, when compared with normal subjects, strongly suggesting that the endplates and vertebral bodies are the sources of pain [29, 30]. These reports suggest that Modic type 1 signal shows an active inflammatory stage [2, 5, 29, 30]. In contrast, type 2 changes were found to be associated with fatty degeneration of the red marrow and its replacement by yellow marrow. Thus, it had been concluded that type 1 changes correspond to the inflammatory stage of DDD and indicate an ongoing active degenerative process, whereas type 2 changes represent the fatty stage of DDD and are related to a more stable and chronic process.

In the study by Toyone et al. [5], 70% of the patients with type 1 Modic changes and 16% of those with type 2 changes were found to have segmental hypermobility, defined as a sagittal translation of 3 mm or more on dynamic flexion-extension films [5]. In a study assessing osseous union following lumbar fusion in 33 patients, Lang et al. found that all 19 patients with solid fusion had type 2 Modic changes, whereas 10 of the 14 patients with nonunion had type 1 changes [31, 32]. They suggested that Modic type 1 in patients with unstable fusions might be related to reparative granulation tissue, inflammation, edema, and hyperemic changes. They concluded that the persistence of type 1 Modic changes after fusion suggests pseudoarthrosis. Similarly, Buttermann et al. observed that nonfusion was associated predominantly with the persistence of type 1 Modic changes [15]. There are patients having very low back pain Modic type 1 and in addition patients with unbearable pain will spend for the failed fusion surgery. For this reason, we performed dynamic stabilization in Modic type 1 and 2 patients.

Hinged screw systems have been used for posterior dynamic stabilization in the current series. The advantages of this system are as follows. (i) These systems stabilize the spine and restore the neutral zone [33–35]. (ii) They provide a simple surgery, when compared with anterior, posterior, or combined fusion surgery. (iii) These types of dynamic systems allow performing lumbar lordosis during the surgery. (iv) Pseudoarthrosis rate is high in cases with fusion surgery [16, 31]. (v) The clinical experience demonstrated good results in the literature [36, 37].

Chataigner et al. studied 56 patients who underwent anterior procedures with bone grafting for LBP [16]. Their best results were obtained in patients with Modic type 1 lesions. The results were poorer in patients who had black discs without endplate involvement or Modic type 2 lesions. Among five nonunions, three requiring posterior revision surgery were observed in Modic type 2 changes. Anterior surgery, with disc herniation associated with Modic type 1 or 2 as the basis for the implementation of changes, is difficult. Because these patients for the treatment of disc herniation and discectomy ago posterior made, then the patients given the same or a different session, the anterior position to apply the anterior fusion surgery. Anterior surgery is time consuming and is an intervention method with a high likelihood of complications. For these patients instead of an application, we propose a posterior dynamic stabilization.

Kwon et al. studied the long-term efficacy of PLIF with a threaded fusion cage based on vertebral endplate changes in DDD [18]. They found that the fusion rate was 80.8% for patients with Modic type 1 changes, 83.6% with Modic type 2 changes, and 54.5% with Modic type 3 changes. Furthermore, the nonfusion rate was 20%. This ratio is higher for patients with Modic type 1 as a high proportion of patients continue to complain about pain and do not see the benefits of treatment. Vital et al. assessed the clinical and radiological outcomes following instrumented posterolateral fusion in 17 patients with chronic LBP and type 1 Modic changes [32]. Six months later, all type 1 changes had converted, with 76.5% being converted to type 2 changes and 23.5% back to normal, and clinical improvement was seen in all patients. They concluded that fusion accelerates the course of type 1 Modic changes probably by correcting the mechanical instability, and that these changes appear to be a good indicator of satisfactory surgical outcome after arthrodesis.

The natural course of the signal anomalies reported by Modic et al. was subsequently followed up by the same authors [2]. Five of the six type 1 lesions were replaced by type 2 signal anomalies over 14–36 months. The type 2 lesions remained stable over 2-3 years of follow-up evaluation. Lang et al. showed that the persistence of Modic type 1 signal after arthrodesis suggests pseudoarthrosis [31]. Toyone et al. concluded that Modic type 1 signal is associated with instability, requiring arthrodesis more commonly than Modic type 2 change, which can accompany nerve-root compromise [5].

In brief, we can state that Modic type 1 changes are associated with instability and painful disorders connected with instability. In such cases, posterior dynamic stabilization could be an effective and alternative treatment modality.

## Figures and Tables

**Table 1:** Results of radiological lumbar lordosis, *α* angle, and intervertebral space (IVS).

	Preop	Postop (3 months)	Postop (12 months)	Postop (24 months)	*P* value
Lumbar lordosis (LL)					
Median	44.85	43.45	43.86	43.56	0.059
Min–max	14–72	18–70	18–71	17–69	
*α* angle					
Median	10.17	9.98	9.93	10.06	0.685
Min–max	1–30	0–33	0–31	2–32	
Intervertebral space (IVS)					
Median	0.28	0.27	0.28	0.28	0.029
Min–max	0-0	0-0	0-0	0-0	

Friedman test (mean and *P* value); Wilcoxon Signed Ranks Test IVS (preop 3 months: *P* < 0.005, preop 12 months: *P* < 0.004, and preop 24 months: *P* < 0.005).

**Table 2:** Comparison of the outcomes of visual analog scale (VAS) and Oswestry Disability Index (ODI) scores in the groups. Both groups exhibited significant reduction in pain over time.

	Mean	Comparison		*P* value
Visual analog scale (VAS)	Preop: 7.20 3 months: 2.70 12 months: 1.53 24 months: 0.95	Preop: 3 months Preop: 12 months Preop: 24 months 3–12 months	3–24 months 12–24 months	0.000

Oswestry Disability Index (ODI)	Preop: 65.90 3 months: 22.80 12 months: 11.10 24 months: 4.94	Preop: 3 months Preop: 12 months Preop: 24 months 3–12 months	3–24 months 12–24 months	0.000

Friedman test (mean and *P* value); Wilcoxon Signed Ranks Test.
